# Catalytic Performance of a Class III Old Yellow Enzyme and Its Cysteine Variants

**DOI:** 10.3389/fmicb.2018.02410

**Published:** 2018-10-12

**Authors:** Anika Scholtissek, Eric Gädke, Caroline E. Paul, Adrie H. Westphal, Willem J. H. van Berkel, Dirk Tischler

**Affiliations:** ^1^Environmental Microbiology Group, Interdisciplinary Ecological Center, Institute of Biosciences, Technical University Bergakademie Freiberg, Freiberg, Germany; ^2^Microbial Biotechnology, Department of Biology and Biotechnology, Ruhr-University Bochum, Bochum, Germany; ^3^Laboratory of Organic Chemistry, Wageningen University and Research, Wageningen, Netherlands; ^4^Department of Biotechnology, Delft University of Technology, Delft, Netherlands; ^5^Laboratory of Biochemistry, Wageningen University and Research, Wageningen, Netherlands

**Keywords:** biocatalysis, ene reductase, flavoprotein, inactivation, actinobacteria, protein engineering, *Rhodococcus opacus* 1CP, cysteine modification

## Abstract

Class III old yellow enzymes (OYEs) contain a conserved cysteine in their active sites. To address the role of this cysteine in OYE-mediated asymmetric synthesis, we have studied the biocatalytic properties of OYERo2a from *Rhodococcus opacus* 1CP (WT) as well as its engineered variants C25A, C25S and C25G. OYERo2a in its redox resting state (oxidized form) is irreversibly inactivated by *N*-methylmaleimide. As anticipated, inactivation does not occur with the Cys variants. Steady-state kinetics with this maleimide substrate revealed that C25S and C25G doubled the turnover frequency (*k*_cat_) while showing increased *K*_M_ values compared to WT, and that C25A performed more similar to WT. Applying the substrate 2-cyclohexen-1-one, the Cys variants were less active and less efficient than WT. OYERo2a and its Cys variants showed different activities with NADPH, the natural reductant. The variants did bind NADPH less well but *k*_cat_ was significantly increased. The most efficient variant was C25G. Replacement of NADPH with the cost-effective synthetic cofactor 1-benzyl-1,4-dihydronicotinamide (BNAH) drastically changed the catalytic behavior. Again C25G was most active and showed a similar efficiency as WT. Biocatalysis experiments showed that OYERo2a, C25S, and C25G converted *N*-phenyl-2-methylmaleimide equally well (81–84%) with an enantiomeric excess (*ee*) of more than 99% for the *R*-product. With cyclic ketones, the highest conversion (89%) and *ee* (>99%) was observed for the reaction of WT with *R*-carvone. A remarkable poor conversion of cyclic ketones occurred with C25G. In summary, we established that the generation of a cysteine-free enzyme and cofactor optimization allows the development of more robust class III OYEs.

## Introduction

**Scheme 1:**
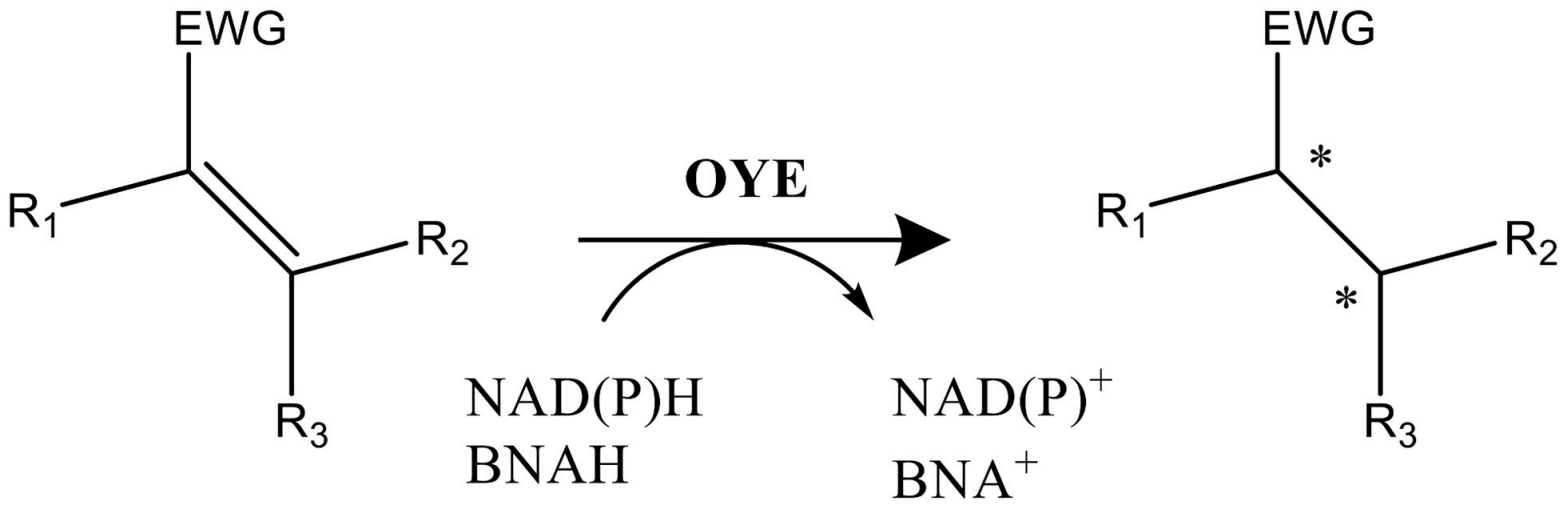
Reaction catalyzed by FMN-containing OYEs. NAD(P)H or BNAH serve as the electron donor and the substrate (α,β-unsaturated alkene) is activated by an electron withdrawing group (EWG; e.g., aldehyde, ketone, or nitro among others). Two potential stereo centers are indicated by an ^*^.

Protein engineering is a powerful tool to improve the catalytic properties of enzymes. Through inducing subtle changes in amino acid side chains, it is possible to optimize binding of specific ligands, thermostability, reaction rates and catalytic efficiency (Balke et al., 2017). Also, the enantioselectivity can be switched (van Den Heuvel et al., 2000). An encouraging enzyme class in the focus of biocatalysis is the flavin-dependent ene reductase (ER) from the Old Yellow Enzyme family (OYEs, EC 1.6.99.1). The FMN-containing OYEs catalyze the selective reduction of activated α,β-unsaturated alkenes yielding valuable alkanes containing one or two chiral carbon centers (Scheme 1). Capable of catalyzing the asymmetric *trans*-hydrogenation of various alkene substrates such as cyclic enones, maleimides, aldehydes, or (di)-carboxylic acids (Swiderska and Stewart, 2006a; Nivinskas et al., 2008; Toogood et al., 2008; Fryszkowska et al., 2009; Gao et al., 2012; Fu et al., 2013; Riedel et al., 2015; Scholtissek et al., 2017b; Toogoood and Scrutton, 2018), ERs promise potential applications in industrial processes due to their exquisite *regio-*, stereo- and enantioselectivity. The FMN cofactor receives electrons from NAD(P)H, but ERs also accept nicotinamide coenzyme biomimetics (NCBs) (Knaus et al., 2016; Scholtissek et al., 2017a).

A detailed summary of protein engineering studies on OYEs was reported earlier (Amato and Stewart, 2015; Toogoood and Scrutton, 2018). OYE1 from *Saccharomyces pastorianus* (Saito et al., 1991) is the most investigated OYE toward mutagenesis so far. Due to the small size of the active pocket, OYE1 is limited to smaller substrates and to a strict enantioselectivity (Swiderska and Stewart, 2006b). Replacement of Trp116 by Ile or Phe led to an opposite binding of several substrates, which induced an inverted stereochemical outcome (Padhi et al., 2009; Pompeu et al., 2013; Walton et al., 2014). Further amino acid replacements of OYE1 conserved residues Tyr196 (Kohli and Massey, 1998), Thr37 (Xu et al., 1999), and His191/Asn194 (Fox and Karplus, 1994), affecting both the oxidative as well as reductive half-reaction.

OYEs from class III (Scholtissek et al., 2017a) were engineered to improve stability or to modulate their reduction potential (Spiegelhauer et al., 2010; Riedel et al., 2015). Introduction of a characteristic salt bridge in OYERo2 generated a protein variant (OYERo2a) with similar catalytic properties as the wildtype enzyme (Riedel et al., 2015). However, thermal stability and tolerance toward organic solvents were highly improved. In subsequent studies, we observed that several substrates, especially maleimides, have an inhibitory effect on OYERo2a (discussed as wild type (WT) here). Considering the presence of a cysteine residue near the *N*-terminus of the protein—only conserved for class III OYEs—it was assumed that this cysteine might form a thioether due to a Michael addition reaction with maleimides (Gregory, 1955). This cysteine was already the subject of a previous study on xenobiotic reductase A (XenA) and was found to modulate the FMN/FMNH^−^ reduction potential (Spiegelhauer et al., 2010). Structural investigations of XenA and two Cys variants revealed that the cysteine residue determines whether the oxidation of NADPH (reduction of FMN) or the reduction of the alkene substrate (oxidation of FMNH^−^) is rate-limiting.

In this study, we produced C25A, C25S, and C25G variants of OYERo2a and studied their kinetic and biocatalytic properties. Interestingly, we found that the Cys replacements result in substrate-dependent catalytic efficiencies and enantioselectivities. Moreover, we established that the natural electron donor NADPH can be cost-effectively replaced by the synthetic cofactor BNAH.

## Experimental

### Chemicals and enzymes

All chemicals and substrates used for buffers and biotransformations were purchased from Sigma-Aldrich (Steinheim, Germany), Carl Roth (Karlsruhe, Germany) and Merck Chemicals GmbH (Darmstadt, Germany) and of the purest grade available. Nicotinamide adenine dinucleotide phosphate (NADPH) was purchased from Prozomix (Northumberland, UK). 1-Benzyl-1,4-dihydronicotinamide (BNAH) was synthesized as described previously (Paul et al., 2013).

### Site-directed mutagenesis, expression, and purification

All plasmids, primers and mutant megaprimers used in this study are presented in Table 1. Site directed mutagenesis of *oyeRo2a* was performed in two steps (Scheme S1, Supplementary Material).

**Table 1:** Plasmids and primers used in this study.

**Plasmids**	**Relevant characteristic(s)**	**Source**
**PLASMIDS**		
pS*Ro*OYE2a_P01	*oyeRo2a* of *R. opacus* 1CP (1.098-kb *Nde*I/*Not*I fragment) cloned into pET16bP	Riedel et al., 2015
pS*Ro*OYE2a_P02	*oyeRo2a_C25S* of *R. opacus* 1CP (1.098-kb *Nde*I/*Not*I fragment) cloned into pET16bP	This study
pS*Ro*OYE2a_P03	*oyeRo2a_C25A* of *R. opacus* 1CP (1.098-kb *Nde*I/*Not*I fragment) cloned into pET16bP	This study
pS*Ro*OYE2a_P04	*oyeRo2a_C25*G of *R. opacus* 1CP (1.098-kb *Nde*I/*Not*I fragment) cloned into pET16bP	This study
**PRIMER**		
C25S_fw	5′-ATGGCTCCTATGTCTCAATACTCAGCAGATG-3′	This study
C25A_fw	5′-ATGGCTCCTATGGCTCAATACTCAGCAGATG-3′	This study
C25G_fw	5′-ATGGCTCCTATGGGTCAATACTCAGCAGATG-3′	This study
pET_check_rev	5′-CAGCTTCCTTTCGGGCTTTGTTAG-3′	Qi et al., 2016
**MEGAPRIMER**		
oyeRo2a_C25S	*oyeRo2a* gene of *R. opacus* 1CP (1.098-kb fragment) containing the substitution guanine77 → cysteine77	This study
oyeRo2a_C25A	*oyeRo2a* gene of *R. opacus* 1CP (1.098-kb fragment) containing the substitutions thymine76 → guanine76 and guanine77 → cysteine77	This study
oyeRo2a_C25G	*oyeRo2a* gene of *R. opacus* 1CP (1.098-kb fragment) containing the substitution thymine76 → guanine76	This study

(1) Synthesis of the mutant megaprimer by template mutation. Single-site mutated *oyeRo2a* genes were amplified by PCR from pET16bp_oyeRo2a DNA solution (100 ng μl^−1^) applying the respective primers (Table 1). The received PCR products served as mutant megaprimers oyeRo2a-C25S, oyeRo2a-C25A and oyeRo2a-C25G. (2) Annealing of the mutated megaprimer (500 ng) and the original pET16bp_oyeRo2a vector (50 ng) using the GeneMorph® EzClone Reaction (Agilent Technologies) for a novel PCR reaction. Add-on DpnI digestion was applied in order to remove the *E. coli* template DNA. Resulting recombinant plasmids pS*Ro*OYE2a_P02, pS*Ro*OYE2a_P03 and pS*Ro*OYE2a_P04 were transformed into *E. coli* BL21 (DE3) pLysS. Expression and purification of the protein variants was performed as described earlier for OYERo2a (Riedel et al., 2015).

### Enzyme activity and kinetic characterization

Specific activities of ERs were determined spectrophotometrically by following the consumption of NADPH or BNAH at 340 nm as described earlier (Riedel et al., 2015). However, the enzyme, buffer and substrate concentrations were slightly modified to achieve best performance. In a pre-screen it turned out that *N*-methylmaleimide served as best substrate to generate reproducible kinetics (data not shown) and thus it was used herein as the major substrate. The molar absorption coefficients 6.22 mM^−1^cm^−1^ and 4.75 mM^−1^cm^−1^ were used for NADPH and BNAH, respectively. Standard assays (1.0 mL) were performed at 25°C in 50 mM phosphate buffer (KH_2_PO_4_/Na_2_HPO_4_; pH 7.1) containing 180 μM NADPH and 1 mM of the respective substrate.

Steady-state kinetic parameters for the electron donor were determined using a fixed concentration of *N*-methylmaleimide (1 mM) and varying the concentration of NADPH or BNAH in a range from 0 to 350 μM. The reaction was started through the addition of enzyme in a final concentration of 36 nM. Steady-state kinetic parameters for the substrate were determined using a fixed concentration of NADPH (180 μM) and varying the concentration of *N*-methylmaleimide in a range from 0 to 350 μM or 2-cyclohexen-1-one in a range from 0 to 20 mM. The reaction was started through the addition of enzyme in a final concentration of 36–72 nM. All activity assays were performed in triplicates and the calculated mean values and respective standard deviation are given as results later.

### Cofactor analysis and enzyme stability

Flavin content of the Cys variants was determined spectrophotometrically from absorption scans (300–600 nm) as described earlier for OYERo2a (Riedel et al., 2015) using the molar absorption coefficient for free FMN (ε_445_ = 12.5 mM^−1^ cm^−1^) (Whitby, 1953). Free flavin was obtained from incubating the protein sample (25 μM) for 20 min at 95°C either in the dark (closed water bath) or under exposure to light (open thermomixer), respectively. After a centrifugation step (20 min; 16,000 × g), a spectral analysis was performed on the supernatant.

Long-time stability of the proteins was investigated at 4°C in 50 mM KH_2_PO_4_/Na_2_HPO_4_ buffer (pH 7.1) using enzyme concentrations of 7–36 μM. Eppendorf tubes, containing the enzyme solution, were kept either in the dark or under exposure to light. Specific activity was followed for 8 days at intervals of 24 h applying the standard assay with 1 mM *N*-methylmaleimide as the substrate.

### Covalent enzyme modification and determination of cysteine content

In order to monitor enzyme inactivation caused by maleimides for OYERo2a as well as mutant proteins, specific activity of untreated enzyme (0.3 mg mL^−1^) was compared with that of enzyme pre-incubated for 120 min in 50 mM KH_2_PO_4_/Na_2_HPO_4_ buffer pH 7.1 at 4°C with 10 mM *N*-methylmaleimide. The specific activity was measured as described above using 1 mM *N*-methylmaleimide as a substrate and pre-incubated or untreated protein in a final concentration of 3 μg mL^−1^.

The Ellman's test was used to quantify the cysteine residues (Ellman, 1959; Riener et al., 2002). The enzymes (2.2 mg mL^−1^) were diluted 1:50 with the working solution (2.5 mM sodium acetate, 0.1 mM DTNB, 8 M urea, 50 mM phosphate buffer pH 7.5) and incubated for 3 min at 20°C. Extinctions were measured at 412 nm (ε_412_ = 14,150 M^−1^ cm^−1^) (Riddles et al., 1979). To obtain a proper blank, OYERo2a and C25S variant (2.2 mg mL^−1^) were pre-incubated with 5 mM *N*-methylmaleimide for 3 h to mask all cysteine residues and extinctions were measured as described above. The measured blank value (corrected for DTNB and for the FAD absorbance of the enzyme) was subtracted from the normal extinction. Cysteine concentration was calculated from the calibration y = 73.942 x, whereas y is the cysteine concentration in μM and x is the extinction. All assays (enzyme samples and blanks) were performed at least 3-times.

### Biotransformation reactions and stereochemistry

Conversion of cyclic enones and maleimides was performed using 1.5-mL sealed glass vials containing the following components in a final volume of 1 mL: 25 mM KH_2_PO_4_/Na_2_HPO_4_ buffer (pH 7.1), 10 mM substrate, 12 mM NADPH, and 2 μM enzyme. BNAH, used as a nicotinamide cofactor mimic, was dissolved in methanol and added in a final concentration of 16.7 mM. Reactions were performed for 4 h at 18°C in vials constantly shaken at 650 rpm in the dark. Extractions followed for 10 min with ethyl acetate (1:2) containing dodecane as an internal standard. Extracts were dried with MgSO_4_ and stored at 7°C prior to gas chromatography (GC) and HPLC analyses.

Product concentrations were calculated based on calibration curve equations using 5 mM dodecane as an internal standard. Enantiomeric excess was measured via GC or HPLC with chiral columns. GC analyses were carried out on a Shimadzu GC-2010 gas chromatograph equipped with an FID on the assigned column (see Table S1). The calibration curves using 5 mM dodecane as an internal standard were linear in the range of product detection (*R*^2^ > 0.99). Authentic samples were used to determine the absolute configuration of the product enantiomers. Specific column information, temperature programs and retention times are listed in the Supplemental Material (Tables S1, S2).

### Structural modeling

An amino acid sequence alignment based on previously published work (Scholtissek et al., 2017a) was used to generate a sub-alignment of class III OYEs. This served as a template for the subsequent modeling efforts. The following sequences with respective available structural data were used to generate dimeric homology models of OYERo2a and Cys variants including FMN in their oxidized forms: TOYE (OYE from *Thermoanaerobacter* sp.; pdb: 3KRZ; Adalbjörnsson et al., 2010), XenA (pdb: 3L5L; Spiegelhauer et al., 2010), and YqjM (ene reductase from *Bacillus subtilis*; pdb: 1Z41; Kitzing et al., 2005). For this, the following tools were employed: MEGA7-mac for the sequence alignment (Kumar et al., 2016), Modeler version 9.15 for comparative homology modeling, and PyMol V1.1r1 for visualization (Sali and Blundell, 1993; Eswar et al., 2006; Riedel et al., 2015).

## Results

### Structural modeling of active site wild-type OYERo2a and Cys variants

**Figure 1:**
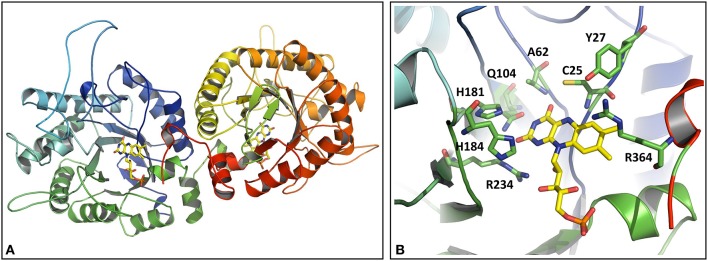
Homology model of OYERo2a. **(A)** Three-dimensional model of the dimeric protein structure. The FMN prosthetic group is indicated in yellow. **(B)** Active site model. The side chains interacting with the flavin are shown in stick models (green) and colored by elements (red = oxygen-containing group; blue = nitrogen containing group). The FMN cofactor is shown as stick model and colored by elements with carbons in yellow. Note that R364 belongs to the adjacent subunit. C25 is in hydrogen bonding distance to the O4-atom of the isoalloxazine ring of the flavin.

The OYERo2a structure was modeled as a dimer according to the template structures of TOYE (3KRU; Adalbjörnsson et al., 2010), XenA (3L5L; Spiegelhauer et al., 2010), and YqjM (1Z41; Kitzing et al., 2005). This is in congruence with our previously made observations from structural modeling as well as from analytical gel filtration of OYERo2a (Riedel et al., 2015). The FMN cofactor was positioned into the active site by using the structure of YqjM (1Z41) as the building template (Figure 1A). A closer look into the active site of OYERo2a (Figure 1B) showed that all catalytically important amino acid residues are in a similar position as in the template structures (Scholtissek et al., 2017a). The class III conserved residue Arg364 points into the active site of the respective adjacent monomer. The mutations introduced at Cys25 did not change the model and thus the active site construction, which is in accordance with results obtained for XenA, where no structural perturbations were observed upon changing Cys25 (Spiegelhauer et al., 2010). However, it is clear that substitution of Cys with Ser at position 25 does nearly perfectly match the OYERo2a structure, while the substitutions with Ala or Gly yields a more open active site.

### Cofactor analysis and stability

**Figure 2:**
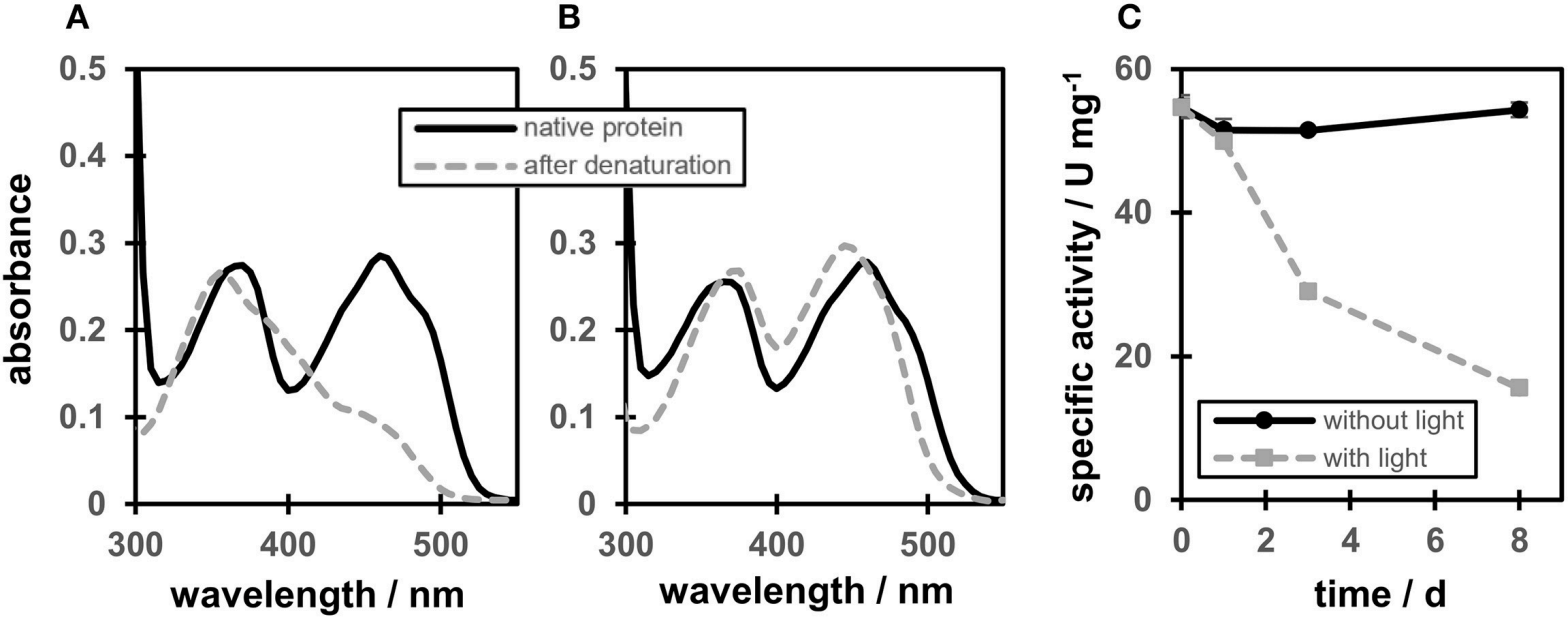
Degradation of the flavin cofactor and stability of OYERo2a (WT). **(A)** OYERo2a (WT) before denaturation (black line) and free flavin after denaturation at 95°C for 20 min while protein was exposed to light. **(B)** OYERo2a (WT) before denaturation (black line) and after denaturation at 95°C for 20 min while stored in a dark water bath. **(C)** Specific activity of OYERo2a over a time interval of 8 days while stored at 4°C in 50 mM KH_2_PO_4_/Na_2_HPO_4_ buffer pH 7.1 in the dark (black line) or under exposure to light (gray line).

A protein-flavin ratio of 1:1 was obtained for all proteins (WT, C25S, C25A, and C25G), which is in analogy to our previous study on OYERo2 (Riedel et al., 2015). RP-HPLC analysis confirmed that the Cys variants contain FMN as prosthetic group. Protein-bound flavin showed two maxima at 370 and 460 nm and also a characteristic shoulder at around 485 nm (Figures 2A,B, black lines). However, depending on the presence of a light source, the flavin cofactor revealed two different spectra after denaturation of the protein at 95°C. When protein denaturation was performed in a thermomixer under exposure to light, a maximum in absorption of the flavin was observed around 355 nm, while a strong decrease in absorption occurred around 450 nm (Figure 2A, gray line). Denaturation in the dark revealed the expected FMN cofactor with maxima at 374 and 446 nm (Figure 2B, gray line).

These observations are in congruence with long time protein stability tests. Incubating the WT protein at 4°C in 50 mM KH_2_PO_4_/Na_2_HPO_4_ buffer pH 7.1 in the dark and determination of the specific activity over a time interval of 8 days, showed no significant activity drop (Figure 2C, black line). However, under exposure to light, only 10% residual activity remained after the same time (Figure 2C, gray line). These observations are referred to a light-mediated photoreduction of the FMN cofactor as it was published before for XenA (Spiegelhauer et al., 2009, 2010). The previous study described photoreduction as a two-step mechanism ensuring single electron transfer. In a first step a red anionic flavin-semiquinone is formed, followed by the formation of a flavin-hydroquinone (Spiegelhauer et al., 2009). However, while XenA was completely reoxidized after exposure to air, OYERo2a and variants could not be regenerated. Instead, using RP-HPLC and as reference the ribityl side-chain lacking lumichrome (Holzer et al., 2005) revealed that under the conditions applied here, OYERo2 bound flavin is degraded to lumichrome.

### Inactivation of WT by covalent binding of maleimides

The amino acid sequence of OYERo2a comprises a single Cys residue at position 25 (Riedel et al., 2015). Quantitative determination of the cysteine residues applying Ellman's test confirmed that the WT enzyme contains one cysteine (ratio of mol cys per mol protein:1.33 ± 0.14) whereas the Cys variants did not possess any cysteine (C25A: 0.21 ± 0.02; C25G: 0.07 ± 0.15; C25S: 0.08 ± 0.09) (see Table S3 in Supplementary Material).

**Figure 3:**
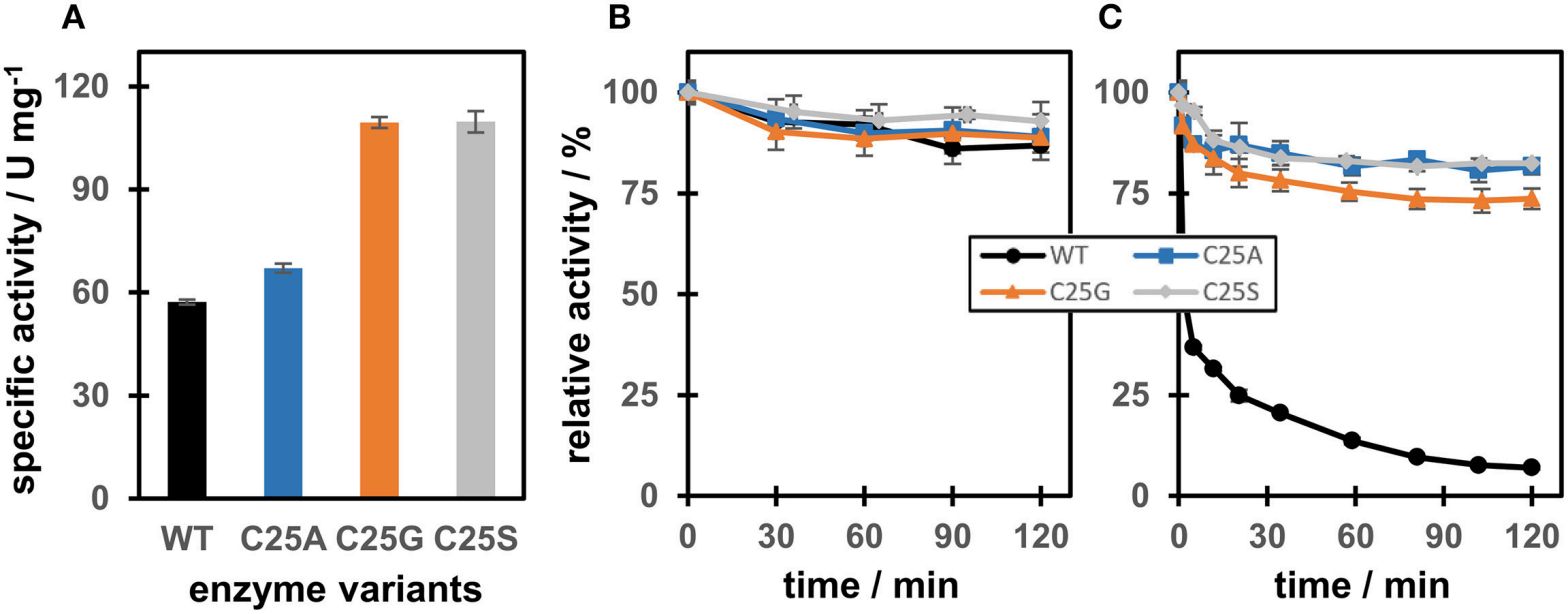
Specific activity and time-dependent inactivation of OYERo2a WT and Cys variants with *N*-methylmaleimide. **(A)** Specific activities using *N*-methylmaleimide as a substrate. **(B)** Relative activity of WT and variants after incubation for 120 min in 50 mM KH_2_PO_4_/Na_2_HPO_4_ buffer pH 7.1 at 4°C (in the dark). Samples were taken at several time points to measure enzyme activity. **(C)** Relative activity of WT and variants after incubation for 120 min with 10 mM *N*-methylmaleimide in 50 mM KH_2_PO_4_/Na_2_HPO_4_ buffer pH 7.1 at 4°C (in the dark). Samples were taken at several time points to measure enzyme activity.

Interestingly, using maleimide as a substrate, the specific activity for the Cys variants was 192% (C25G), 191% (C25S), and 117% (C25A) of that of OYERo2a (Figure 3A). When the enzyme was incubated over 2 h in the dark, relative activity remained between 90 and 100% for each variant (Figure 3B). However, when the enzymes were pre-incubated with 10 mM *N*-methylmaleimide for 120 min, the activity of the Cys variants remained stable (80–100% relative activity), while the OYERo2a became almost completely inactivated (Figure 3C). This inactivation of the enzyme is a strong indication that Cys25 plays an important role in the binding of OYERo2a substrates.

### Steady-state kinetics

#### Cofactor preference

**Figure 4:**
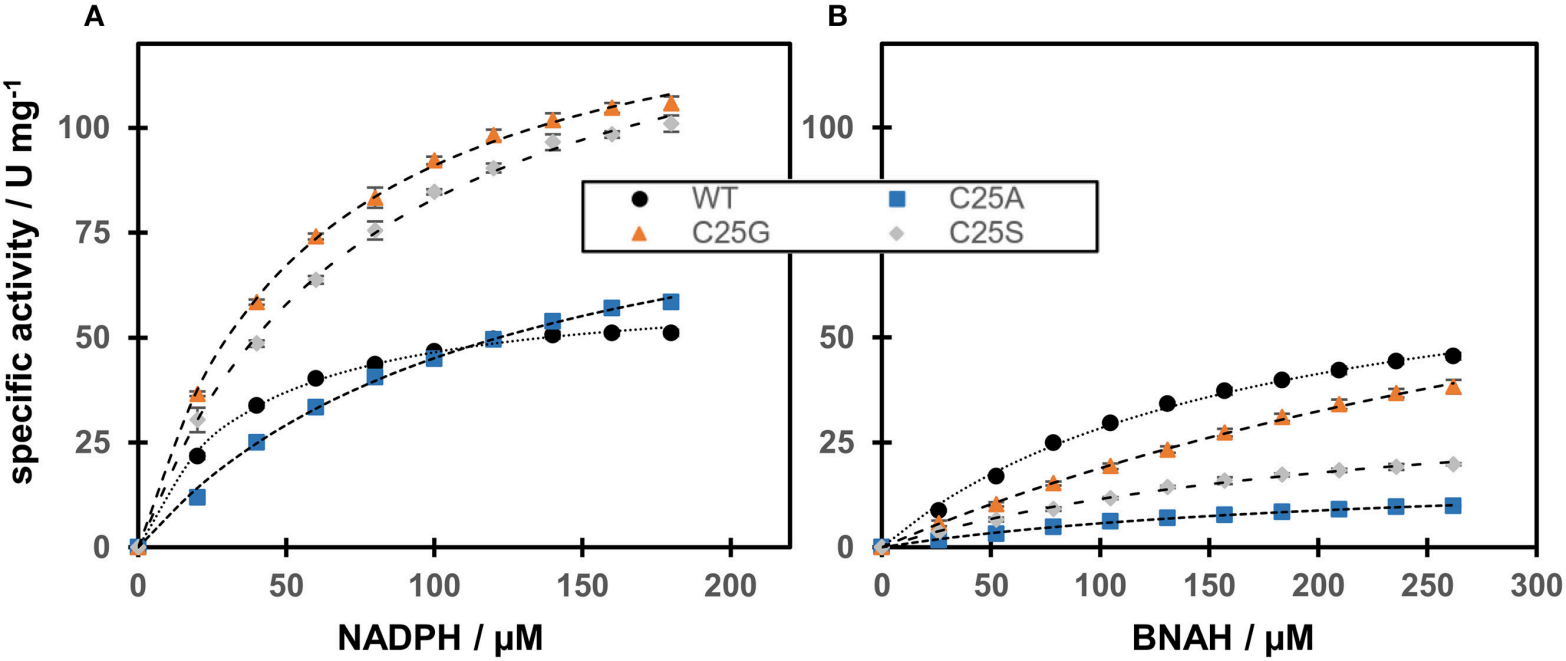
Steady-state kinetics of OYERo2a (WT) and Cys variants regarding cofactor preference. The assay contained different amounts of nicotinamide cofactors NADPH **(A)** and BNAH **(B)**. The Michaelis-Menten model has been used to fit the data.

The activity of OYERo2a and Cys variants with the natural nicotinamide cofactor NADPH was compared with that of the synthetic nicotinamide cofactor BNAH using *N*-methylmaleimide as the fixed substrate (Figure 4). The OYERo2a and Cys variants followed Michaelis-Menten kinetics and respective kinetic parameters were determined. From Table 2 it can be noticed that OYERo2a has similar *k*_cat_ values for NADPH and BNAH. However, the Cys variants have much lower *k*_cat_ values with BNAH, while a remarkable increase is observed with NADPH. An exception represents C25G which has a high turnover frequency with both NADPH and BNAH. Nevertheless, the catalytic efficiencies (*k*_cat_/*K*_m_ values) of OYERo2a and Cys variants with NADPH are in the same range. This is due to a lower *K*_m_^NADPH^ value (34.8 μM) for OYERo2a compared to the variants (55–121 μM). For all four proteins the catalytic efficiency is higher when using NADPH. However, with OYERo2a the catalytic efficiency ratio between NADPH and BNAH is 2.5:1, while with each of the three variants the ratio is at least 10:1. Highest catalytic efficiency was shown for C25G using NADPH. To conclude, the Cys variants have a strong preference for NADPH over BNAH.

**Table 2:** Steady-state kinetic parameters of OYERo2a (WT) and Cys variants regarding cofactor preference.

	**WT**		**C25S**		**C25A**		**C25G**	

	**NADPH**	**BNAH**	**NADPH**	**BNAH**	**NADPH**	**BNAH**	**NADPH**	**BNAH**
*V*_max_ (U mg^−1^)	62.6 ± 1.0	76.3 ± 2.8	147.0 ± 3.2	38.9 ± 2.3	99.5 ± 3.7	18.9 ± 0.8	141.1 ± 2.1	116.4 ± 7.4
*K*_m_ (μM)	34.8 ± 2.0	169.0 ± 12.4	76.9 ± 4.1	238.7 ± 24.3	120.7 ± 8.7	230.2 ± 17.8	55.2 ± 2.3	516.9 ± 45.0
*k*_cat_ (s^−1^)	44.6 ± 0.7	54.3 ± 2.0	104.6 ± 2.3	27.7 ± 1.6	70.8 ± 2.6	13.4 ± 0.6	100.4 ± 1.5	82.8 ± 5.3
*k*_cat_/*K*_m_ (μM^−1^ s^−1^)	1.28	0.32	1.36	0.12	0.59	0.06	1.82	0.16

*Assays were performed in triplicates. Data (mean values and standard deviation) were analyzed by means of Kaleidagraph (Synergy Software). The given values are calculated from the fits of Michaelis-Menten kinetics following either NADPH or BNAH consumption according to Figure 4. The standard error derived of the best fit is provided*.

#### Substrate dependence

**Figure 5:**
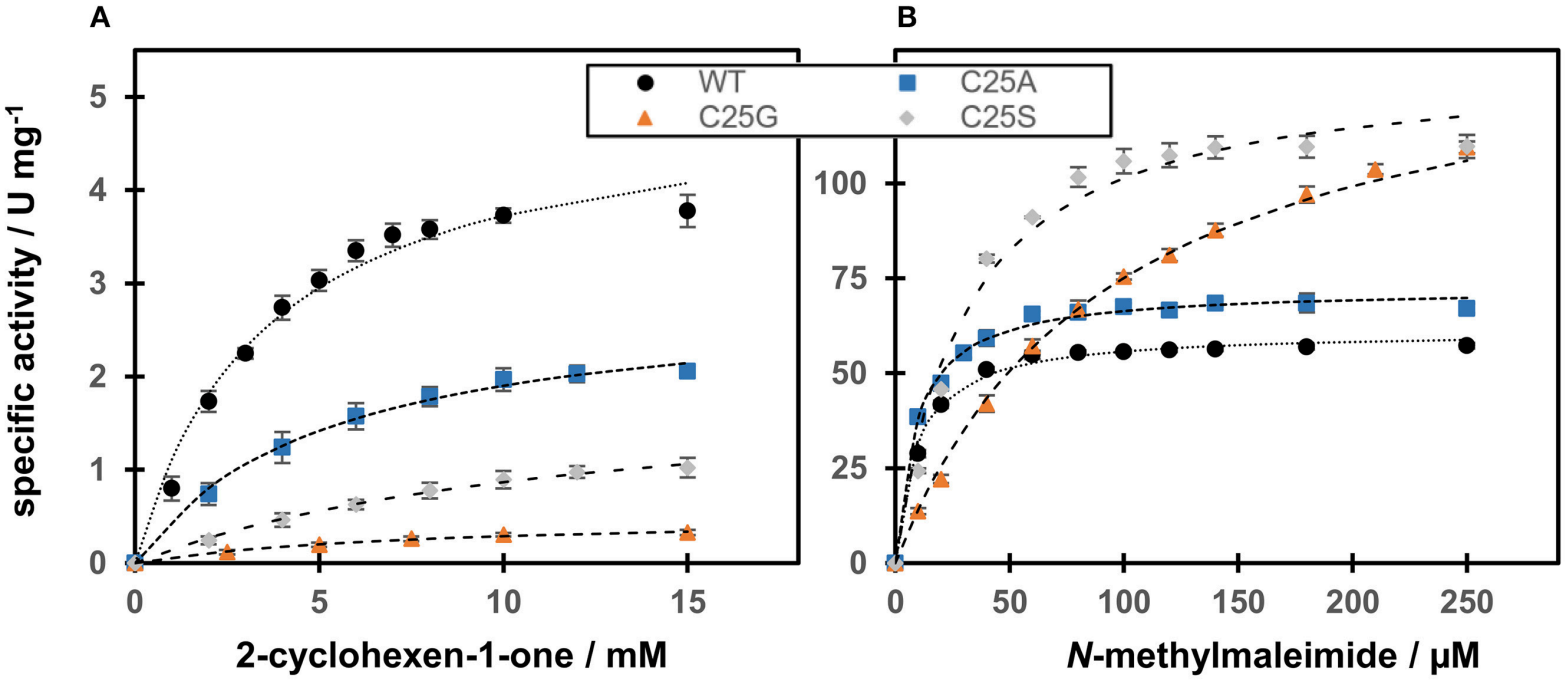
Steady-state kinetics of OYERo2a WT and Cys variants regarding substrate dependence. The assay contained excess NADPH and different amounts of the substrates **(A)** 2-cyclohexen-1-one or **(B)** *N*-methylmaleimide. The Michaelis-Menten model has been used to fit the data.

The catalytic efficiency of WT and Cys variants with *N*-methylmaleimide and 2-cyclohexen-1-one as substrates was studied using NADPH as the fixed co-substrate (Figure 5).

Regarding *N-*methylmaleimide, the apparent *k*_cat_ of WT is comparable with the turnover frequency of C25A (Figure 5A). Both these enzymes share a low *K*_m_ (around 10 μM), resulting in a high catalytic efficiency in the range of 4600–5700 mM^−1^s^−1^ (Table 3). Compared to WT and C25A, C25S has a doubled *k*_cat_ with *N-*methylmaleimide but a tripled *K*_m_ (30 μM) resulting in a lower catalytic efficiency (Table 3). C25G shows the highest *K*_m_ for *N*-methylmaleimide, and therefore a catalytic efficiency 4 times lower than that of WT (Table 3).

**Table 3:** Steady-state kinetic parameters of OYERo2a WT and Cys variants regarding substrate dependence.

	**WT**	**C25S**	**C25A**	**C25G**
***N-*****METHYLMALEIMIDE**				
*V*_max_ (U mg^−1^)	61.0	132.2	72.3	145.9
*K*_m_ (μM)	9.6	30.4	9.1	94.4
*k*_cat_ (s^−1^)	42.3	91.7	50.1	101.2
*k*_cat_/*K*_m_ (μM^−1^ s^−1^)	4.4	3.0	5.5	1.1
coupling efficiency (%)^*^	97.4	89.9	72.7	100.0
**2-CYCLOHEXEN-1-ONE**				
*V*_max_ (U mg^−1^)	5.0	1.6	2.9	0.5
*K*_m_ (mM)	3.5	8.9	5.2	6.2
*k*_cat_ (s^−1^)	3.5	1.1	2.0	0.3
*k*_cat_/*K*_m_ (mM^−1^ s^−1^)	1.0	0.12	0.38	0.05

The values are calculated from the fits of Michaelis-Menten kinetics according to Figure 5. All assays were performed in triplicates and the values have a maximal error of 10% (standard deviation).

* *The coupling efficiency (percentage of alkene reduced per NADPH consumed) is calculated from the NADPH-oxidation (see Table 2) and N-methylmalemide-reducing activity*.

With respect to 2-cyclohexen-1-one, WT showed the highest catalytic efficiency (Figure 5B). However, compared to *N*-methylmaleimide the catalytic efficiencies of all four proteins are reduced by a factor of 400 (WT) to 2500 (C25S) (Table 3). This is due to 70–520 times increase of the Michaelis constant and a 10–340 times decrease of the turnover frequency.

### Biocatalysis applying NADPH and BNAH as electron donors

Regarding conversion rates and enantioselectivities, 2-methyl-*N*-phenylmaleimide **1** as well as *R*- and *S*-carvones (**6** and **7**) are preferred substrates for all four enzymes (Table 4). Especially maleimide-like substrates seem to be favored in terms of kinetics (Table 3) since they show a higher specific activity and efficient coupling of NADPH-consumption with ene reduction. Application of NADPH or BNAH (done for **1** and **2**) showed no significant differences, neither in conversion nor in stereochemistry. Comparing WT with the three protein variants brought no significant differences for substrate **1**. For ketoisophorone **5** and 2-methyl-cyclopentenone **3**, WT enzyme and C25A variant generally performed best. C25G variant showed very low conversions on all substrates, except of 2-methyl-*N*-phenylmaleimide **1**. Because C25G is highly active with *N*-methylmaleimide, but poorly active with cyclic ketones, it appears that residue 25 is critical for determining the substrate specificity of OYERo2 catalysis. This effect was shown with C25G variants of *Rm*OYE and *Ts*OYE where lower conversions were found for *R*-carvone with respect to WT (Nett et al., 2017).

**Table 4:** Conversion and stereochemistry of OYERo2a (WT) and Cys variants with α,β-unsaturated alkenes.

**Substrate**	**WT**		**C25S**		**C25A**		**C25G**	

	**Conversion**	**ee**	**Conversion**	**ee**	**Conversion**	**ee**	**Conversion**	**ee**
	**[%]**	**(%)**	**[%]**	**(%)**	**[%]**	**(%)**	**[%]**	**(%)**
**1** 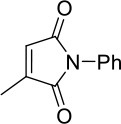	72/84 (NADPH/BNAH)	>99/>99 (*R*)	71/81	>99/>99 (*R*)	65/60	>99/>99 (*R*)	87/81	>99/>99 (*R*)
**2** 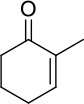	64/49 (NADPH/BNAH)	>99/99 (*R*)	27/17	>99/99 (*R*)	n.d./27	n.d./95 (*R*)	n.d./1	n.a.
**3** 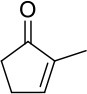	19	>99 (*S*)	4	>99 (*S*)	30	91 (*S*)	< 1	n.a.
**4** 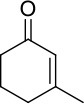	< 1	n.a.	< 1	n.a.	n.d.	n.d.	n.d.	n.d.
**5** 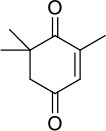	26	93 (*R*)	2	82 (*R*)	34	65 (*R*)	1	71 (*R*)
**6** 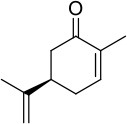	89	>99 (*R*)	70	>99 (*R*)	77	>99 (*R*)	6	>99 (*R*)
**7** 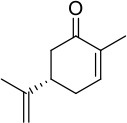	78	96 (*R*)	30	93 (*R*)	n.d.	n.d.	n.d.	n.d.

*n.d. not determined; n.a. not accessible due to low conversion or lack of a chiral center; value < 1 = no observable peak detected. All assays were performed in triplicates and the standard deviation was less than 5% regarding the conversions*.

## Discussion

An *N*-terminal cysteine residue was found to function in the binding of the FMN cofactor of class III OYEs thereby modulating the flavin reduction potential (Spiegelhauer et al., 2010). Additionally, cysteine residues are known for Michael addition reactions with maleimides, forming thioethers (Gregory, 1955). To study its role in further detail, the cysteine residue of OYERo2a was replaced by alanine (A), glycine (G) or serine (S) through site-directed mutagenesis.

Structural modeling of OYERo2a indicated that the replacement of Cys25 is not inducing a general structural rearrangement of the active pocket. However, while Ser isosterically replaces the Cys, more space is created when Ala or Gly is inserted. These three variants (C25 substitutions) were successfully generated and confirmed by gene sequencing as well as by the Ellman's test. The substitutions of Cys25 increased the initial rates with *N*-methylmaleimide, but gave rise to somewhat higher *K*_m_ values for C25S and C25G. As a result, catalytic efficiencies decreased by about 32% (C25S) and 75% (C25G), respectively. Due to a comparable Michaelis constant and a slightly increased turnover frequency, replacement with alanine gave a catalytic efficiency increase of about 25%. However, it seems that the cysteine plays an important role for catalysis of the substrate 2-cyclohexen-1-one leading to the hypothesis that 2-cyclohexen-1-one is structurally closely related to the natural substrate of class III OYEs. The results with 2-cyclohexen-1-one suggest that Cys25 is important for substrate coordination and positioning properly with respect to the FMN cofactor since this residue is an essential part of the substrate binding pocket interacting not only with the O4-atom of the FMN cofactor but also with Tyr27 (Kitzing et al., 2005; Spiegelhauer et al., 2010).

In contrast to the WT enzyme, the variants C25A, C25G, and C25S did not show a time-dependent inactivation with *N*-methylmaleimide in the oxidized resting state (Figure 3). The covalent modification reaction with this maleimide was completely prevented when Ser/Ala/Gly protein variants were applied. Because this property is of benefit for biocatalytic applications, we addressed the catalytic performance of the OYERo2a Cys variants. It appeared that, under the conditions applied, the active site cysteine is not affecting the degree of conversion of *N*-phenyl-2-methylmaleimide. For conversion of 2-cyclohexen-1-one, the cysteine is required. For all other cyclic ketones tested, the WT is also favored, since conversion yield and enantioselectivity is better in most cases.

Besides ene-reducing activity, also the cofactor-dependent FMN reduction needs to be discussed for WT and Cys variants. The following observations can be summarized from this study. WT does most efficiently bind NADPH as well as BNAH, but the variants show an increased turnover frequency only with NADPH. Thus more flexibility of the protein structure might yield a higher turnover frequency for the initial reductant (here NADPH) but this not necessarily leads to a higher ene-reductase activity (Table 3). Only in case of *N*-methylmaleimide a highly efficient transfer of electrons was achieved, which is expressed as coupling efficiency. Therefore, it can be reasoned that the mutual orientation of both cofactor and substrate with respect to the flavin influence the overall catalysis as it was discussed above (Kitzing et al., 2005; Spiegelhauer et al., 2010). With respect to NADPH/BNAH turnover the C25G variant was most efficient and in combination with above mentioned catalytic properties it may be a valuable starting point to further evolve succinimide producing biocatalysts. Especially, this variant showed the highest conversion rates of the pro-chiral *N*-phenyl-2-methylmaleimide at highest enantiomeric excess (Table 4). Further, the BNAH cofactor mimic can replace effectively NADPH in this biotransformation.

Regarding cofactor choice, catalytic efficiency with BNAH is lower in all cases compared to NADPH. Despite that, BNAH seems a reasonable alternative electron donor for OYERo2a biotransformations since it has no inhibitory effects and compared to the natural cofactor NADPH it is more economical (Paul et al., 2013, 2014).

So far, we did not study the operational stability of the enzyme (incubations with gram amounts of substrate and a cofactor-regenerating system, using an immobilized form of the enzyme). We only looked at the inactivation of the oxidized enzyme (the resting state). Nevertheless, this study is a valuable indication to make use of the Cys variants when upscaling the reaction for maleimides for industrial purposes.

## Conclusion

An *N*-terminal cysteine residue, occurring only in class III OYEs, was found to be involved in the catalytic functioning of OYERo2a. We show that the substrate *N*-methylmaleimide inactivates OYERo2a through covalent modification. The cysteine-lacking variants C25S and C25G are not inactivated by *N*-methylmaleimide and show high specific activities with this substrate (up to 147 U mg^−1^) using NADPH as a cofactor.

Interestingly, the choice of cofactor seems to play a major role in OYERo2a catalysis. More precisely, an efficient catalysis was observed for OYERo2a with the cofactor mimic BNAH and *N*-methylmaleimide as the pyrrole-dione substrate.

## Author contributions

AS carried out the cloning and site-directed mutagenesis. Spectral analysis, kinetic characterization (data acquisition and analysis), stability analysis and inhibition studies were established and carried out by EG, AW, and AS. Substrate specificity, product analysis and stereochemistry was carried out by EG and CP. DT carried out the structural modeling. AS, CP, AW, WB, and DT drafted the manuscript, which was critically revised by all authors. All authors read and approved the final manuscript.

### Conflict of interest statement

The authors declare that the research was conducted in the absence of any commercial or financial relationships that could be construed as a potential conflict of interest.
