# Supplementary material for: Catalytic Performance of a Class III Old Yellow Enzyme and Its Cysteine Variants
Source: Front Microbiol. 2018 Oct 12;9:2410. doi: 10.3389/fmicb.2018.02410 (PMC6194350; doi:10.3389/fmicb.2018.02410)
Supplement: Supplementary file 1 [file Data_Sheet_1.docx]

***Supplementary Material***

**Catalytic performance of a class III Old Yellow Enzyme and its cysteine variants**

**Anika Scholtissek^1^, Eric Gädke^1,2^, Caroline E. Paul^3,4^, Adrie H. Westphal^5^, Willem J.H. van Berkel^5^ and Dirk Tischler^2,*^**

^1^Interdisciplinary Ecological Center, Institute of Biosciences, Environmental Microbiology Group, Technical University Bergakademie Freiberg, 09599 Freiberg, Germany

^2^Microbial Biotechnology, Department of Biology and Biotechnology, Ruhr-University Bochum, 44780 Bochum, Germany

^3^Laboratory of Organic Chemistry, Wageningen University & Research, 6708 WE Wageningen, The Netherlands

^4^Department of Biotechnology, Delft University of Technology, 2629 HZ Delft, The Netherlands

^5^Laboratory of Biochemistry, Wageningen University & Research, 6708 WE Wageningen, The Netherlands

*** Correspondence:** Dirk Tischler, Laboratory of Microbial Biotechnology, Department of Biology and Biotechnology, Ruhr-University Bochum, 44780 Bochum, dirk-tischler@rub.de

1. **Supplementary Scheme**

**
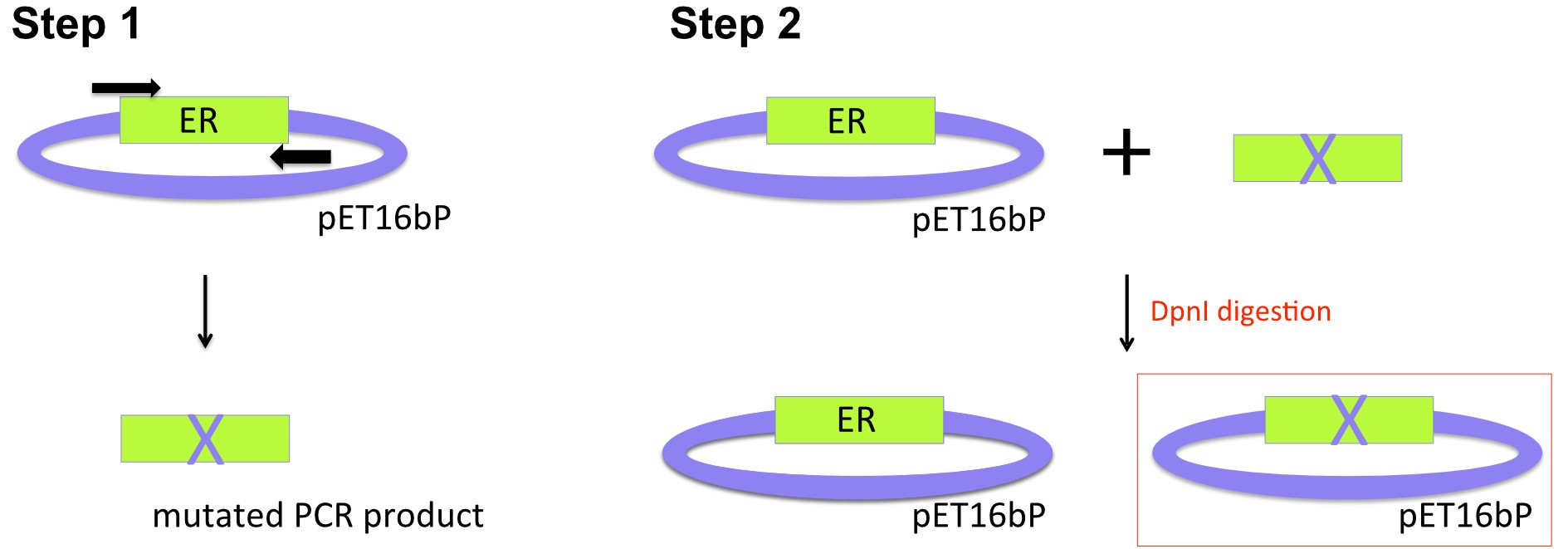
**

Scheme S1. Site directed mutagenesis of *oyeRo2a*. Step 1: Synthesis of the mutant megaprimer. Step 2: Synthesis of the mutated PCR product containing the mutated ene reductase (ER) gene and the template vector.

1. **Supplementary Tables**

**GC analysis**

In the following tables we give detailed information for stereochemistry analysis of substrate conversions applied in the study. The oven temperature programs of the applied columns A, B, C and D as well as the obtained retention times of the alkenes and their hydrogenated products are listed in Table S1. Retention times from HPLC analysis are listed in Table S2. Table S3 shows the detailed values for cysteine concentrations determined from Ellman’s test.

The conversion of substrate to product was measured by gas chromatography (GC). Product concentrations were calculated based on calibration curve equations using 5 mM dodecane as an internal standard. Enantiomeric excess was measured by GC or HPLC with chiral columns. GC analyses were carried out on a Shimadzu GC-2010 gas chromatograph equipped with an FID on the designated column. Authentic samples were used to determine the absolute configuration of the product enantiomers.

Column **A**: CP-Sil 5 CB (50 m × 0.53 mm × 1.0 µm); column flow: 20 mL/min

Column **B**: FS-Lipodex E (50 m × 0.25 mm × 0.12 µm); column flow: 2.12 mL/min, linear velocity: 36.9 cm/sec, split ratio: 50.

Column **C**: CP-Chirasil-Dex CB (25 m × 0.32 mm × 0.25 µm); **2-methylcyclohexenone:** column flow: 1.52 mL/min, linear velocity: 30 cm/sec; split ratio: 150. **ketoisophorone:** column flow: 1.03 mL/min, linear velocity: 22.6 cm/sec; split ratio: 30. **3-methylcyclohexenone:** column flow: 1.59 mL/min, linear velocity: 30 cm/sec; split ratio: 150.

Column **D**: FS-Hydrodex β-TBDAc (50 m × 0.25 mm × 0.25 µm); column flow: 2.23 mL/min, linear velocity: 38.0 cm/sec, split ratio: 100.

**Table S1.** GC column oven programs and retention times for substrates and products.

| **Column** | **Program*^a^*** | **Compound** | **Retention time (min)** |
| --- | --- | --- | --- |
| **A** | 120/2/10/320/2 | 2-methyl-*N*-phenylmaleimide  (*R*,*S*)-2-methyl-*N*-phenylsuccinimide | 8.8  9.5 |
| **A** | 90/5/10/320/2 | cinnamic acid | 12.4 |
| **B** | 80/2/5/110/5/5/130/5/20/220/1 | (-)-(*R*)-carvone  (+)-(2*R*,5*R*)-dihydrocarvone | 18.4  15.6 |
| **B** | 80/2/5/110/5/5/130/5/20/220/1 | (+)-(*S*)-carvone  (-)-(2*R*,5*S*)-dihydrocarvone  (+)-(2*S*,5*S*)-dihydrocarvone | 18.4  16.4  15.6 |
| **C** | 90/11.2/20/130/1/25/225/1 | 2-methylcyclohexenone  (*S*)-2-methylcyclohexanone  (*R*)-2-methylcyclohexanone | 11.1  9.4  9.6 |
| **C** | 110/4/5/130/5/20/220/1 | ketoisophorone  (*R*)-levodione  (*S*)-levodione | 10.8  12.2  12.7 |
| **C** | 70/10/5/80/5/5/90/5/20/220/1 | 3-methylcyclohexenone  (*R*)-3-methylcyclohexanone*^b^*  (*S*)-3-methylcyclohexanone*^b^* | 26.4  19.6 |
| **D** | 80/5/5/100/13/5/130/5/20/230/1 | 2-methylcyclopentenone  (*S*)-2-methylcyclopentanone*^b^*  (*R*)-2-methylcyclopentanone*^b^* | 27.6  20.9  18.4 |

*^a^* GC program: initial temperature (°C) / time (min) / slope (°C)/min / temperature (°C) / slope (°C)/min / temperature (°C) / time (min). *^b^* absolute configuration not confirmed.

**HPLC analysis**

HPLC analyses were performed on a Daicel Chiralcel OD column (25 cm × 0.46 cm) with *n*-heptane/2-propanol (95:5) at 39 °C, with a flow of 1 mL/min.

Table S2. HPLC retention times.

| **Compound** | **Retention time [min]** |
| --- | --- |
| 2-methyl-*N*-phenylmaleimide | 10.5 |
| (*R*)-2-methyl-*N*-phenylsuccinimide | 23.0 |
| (*S*)-2-methyl-*N*-phenylsuccinimide (2b) | 26.6 |

**Determination of cysteine concentration**

Ellman’s test was performed at least 3-times for each enzyme with an enzyme concentration of 1 μM and an average value was determined. Enzymes were pre-incubated with 5 mM *N*-methylmaleimide to mask existing thiol groups. The measured blank value (corrected for DTNB and for the FAD absorbance of the enzyme) was subtracted from the normal extinction. Cysteine concentration was calculated from the calibration y = 73.942 x, whereas y is the cysteine concentration in μM and x is the extinction.

Table S3. Cysteine concentration determined by Ellman’s test.

| **Enzyme** | **Extinction** | **Extinction minus blank value** | **Calculated cysteine concentration** |
| --- | --- | --- | --- |
| WT | 0.0322 | 0.0180 | 1.33 ± 0.14 |
| C25A | 0.0140 | 0.0029 | 0.21 ± 0.02 |
| C25G | 0.0121 | 0.0010 | 0.07 ± 0.15 |
| C25S | 0.0122 | 0.0011 | 0.08 ± 0.09 |
